# Limitations of 16S rRNA Gene Sequencing to Characterize *Lactobacillus* Species in the Upper Genital Tract

**DOI:** 10.3389/fcell.2021.641921

**Published:** 2021-07-29

**Authors:** Jessica L. O’Callaghan, Dana Willner, Melissa Buttini, Flavia Huygens, Elise S. Pelzer

**Affiliations:** ^1^Faculty of Health, Centre for Immunology and Infection Control, Queensland University of Technology, Brisbane, QLD, Australia; ^2^Faculty of Health, School of Biomedical Sciences, Queensland University of Technology, Brisbane, QLD, Australia; ^3^Australian Centre for Ecogenomics, University of Queensland, St Lucia, QLD, Australia; ^4^Department of Computer Science, College of William & Mary, Williamsburg, VA, United States; ^5^The Wesley Hospital, Auchenflower, QLD, Australia

**Keywords:** *Lactobacillus*, genital tract, 16S rRNA, next-generation sequencing, low-biomass

## Abstract

The endometrial cavity is an upper genital tract site previously thought as sterile, however, advances in culture-independent, next-generation sequencing technology have revealed that this low-biomass site harbors a rich microbial community which includes multiple *Lactobacillus* species. These bacteria are considered to be the most abundant non-pathogenic genital tract commensals. Next-generation sequencing of the female lower genital tract has revealed significant variation amongst microbial community composition with respect to *Lactobacillus* sp. in samples collected from healthy women and women with urogenital conditions. The aim of this study was to evaluate our ability to characterize members of the genital tract microbial community to species-level taxonomy using variable regions of the 16S rRNA gene. Samples were interrogated for the presence of microbial DNA using next-generation sequencing technology that targets the V5–V8 regions of the 16S rRNA gene and compared to speciation using qPCR. We also performed re-analysis of published data using alternate variable regions of the 16S rRNA gene. In this analysis, we explore next-generation sequencing of clinical genital tract isolates as a method for high throughput identification to species-level of key *Lactobacillus* sp. Data revealed that characterization of genital tract taxa is hindered by a lack of a consensus protocol and 16S rRNA gene region target allowing comparison between studies.

## Introduction

Molecular microbiology techniques have changed our ability to identify microbial communities, revolutionizing the way we assess female genital tract microbiomes. In cultivation-dependent studies, greater than 95% of the vaginal microbiota in healthy women was classified as lactobacilli (Klebanoff et al., 1991). The advent of cultivation-independent technology platforms provided evidence to suggest that in up to two-thirds of healthy women, the lactobacilli were co-aggregated with a diverse group of microbial community members, and in some cases did not dominate (Ravel et al., 2011; Fettweis et al., 2012). Lactobacilli establish niche dominance through co-aggregation, competitive inhibition, production of metabolic acids, and antimicrobial compounds including bacteriocins (Amabebe and Anumba, 2018). The discovery that lactobacilli do not dominate the lower or upper genital tract of all healthy women and are present in both health and disease suggests that there is redundancy in function and protection based on community membership; and all lactobacilli may not provide the same level of protection in the genital tract environment. This discovery, therefore, casts doubt over the long-held view that a healthy female genital tract is characterized by a *Lactobacillus* sp.-dominant microbiota. The ability to confidently assign lower order taxonomic classification to lactobacilli is critical in advancing our understanding of the protective role played by the various species within this genus in reproductive health. The objective of this study was to examine the discriminatory power of molecular microbiology techniques that exploit the conserved 16S rRNA gene for identification of genital tract lactobacilli. To achieve this goal, the analysis was completed with re-analyzed published data and work in our laboratory.

## Materials and Methods

A basic flowchart of the methods including in this study can be found as Supplementary Figure 1.

### Patient Cohort, Sample Collection, and Genomic DNA Preparation

Samples were collected from women undergoing operative hysteroscopy and laparoscopy. Paired endometrial curettings and endocervical swabs were obtained aseptically from 56 women at the time of surgery. The vagina and perineum were disinfected with povidone iodine at the time of anesthetic induction. An endocervical swab (Amies Agar Transport Swab, Copan) was collected prior to cervical dilatation, followed by disinfection of the ectocervix prior to collection of endometrial curettings and then collection of a second endocervical swab. Specimens were refrigerated at the time of collection and processed within 1 h.

DNA was extracted from 1 mL aliquots of solutions removed from the transport swabs. Endometrial curettings were suspended in 1.5 mL of sterile physiological saline, from which 1 mL was taken for DNA extraction. The remaining samples were stored frozen at −80°C. DNA was extracted using the QiAMP Mini DNA extraction kit (Qiagen, Australia) as per the manufacturer’s instructions with the addition of a preliminary enzymatic lysis step and final elution in 50 μL of sterile water.

Clinical sample cohorts were constructed as previously described (Pelzer et al., 2018a). Briefly, endometrial curettings were fixed and stained with hematoxylin and eosin for menstrual staging and pathology assessment by the pathology department at Sullivan Nicolaides Pathology. The phase of the menstrual cycle at the time of surgery was reported for each patient. The patient samples from all 12 sample types (Table 1) were combined for the below analysis. Genomic DNA was extracted from individual samples prior to pooling an equal volume of DNA from each individual sample.

**TABLE 1:** Sample abbreviations and clinical numbers.

Sample abbreviations	Clinical patients (n)^a^	Full sample name
DGC	8	Dysmenorrhea progestin effect endocervix
DGE		Dysmenorrhea progestin effect endometrium
DPC	13	Dysmenorrhea proliferative endocervix
DPE		Dysmenorrhea proliferative endometrium
DSC	11	Dysmenorrhea secretory endometrium
MGC	4	Menorrhagia progestin effect endocervix
MGE		Menorrhagia progestin effect endometrium
MPC	7	Menorrhagia proliferative endocervix
MPE		Menorrhagia proliferative endometrium
MSC	10	Menorrhagia secretory endocervix
MSE		Menorrhagia secretory endometrium
VIC	3	Virgo intacta

*^*a*^Each patient contributed both an endocervix sample and endometrium sample.*

### Details of Ethical Approval

All patients recruited for this study provided written informed consent. Ethical approval was obtained from the review boards of UnitingCare Health (approval number 2013.04.75), Human Research Ethics Committee and Queensland University of Technology Human Ethics Committee (approval number 1400000686). All methods in this study were performed in accordance with the relevant guidelines and regulations as dictated by these ethics boards.

### Next-Generation Sequencing

**FIGURE 1:**
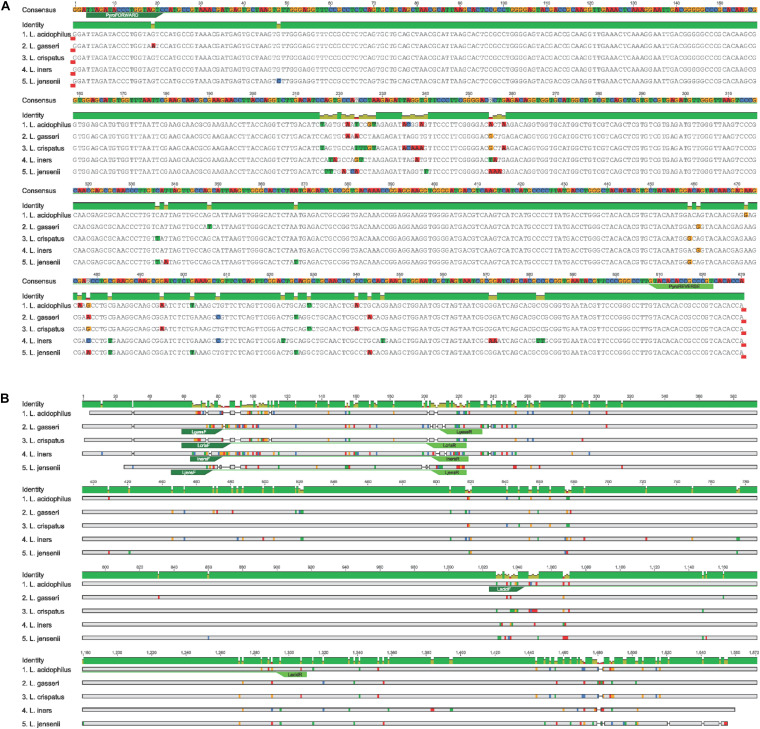
**(A)** Annealing site of NGS 16S rRNA primers to V5-V8 region in the *Lactobacillus* species. **(B)** *Lactobacillus* species-specific primers annealing sites used for qPCR amplification.

The 16S rRNA gene PCR assay was performed using the previously published primers, 803F (5′-ATTAGATACCCTGGTAGTC-3′) and 1392R (5′-ACGGGCGGTGTGTRC-3′) and PCR cycling conditions (Willner et al., 2014). Fusion primers with 454 adaptor sequences were ligated to the 803F and 1392R primers to amplify the V5 and V8 regions of the 16S rRNA gene (Willner et al., 2014). PCR reactions were performed as previously described (Pelzer et al., 2018a). The five frequently encountered genital tract *Lactobacillus* sp. gene sequences were aligned to each other using sequences download from the SILVA database^1^ to determine the degree of variation within the V5-V8 regions of the 16S rRNA gene. The annealing site of the sequencing primers is marked on the alignment (Figure 1A).

### *Lactobacillus* sp.-Specific Quantitative Real-Time PCR

Quantitative real-time PCR assays were performed using previously published primer pairs (Table 2) and cycling conditions. A standard curve was generated using *L. gasseri* ATCC strain 19992. Primer annealing was confirmed using species-specific alignment of the five *Lactobacillus* sp. interrogated in this study (Figure 1B).

**TABLE 2:** 16S rRNA *Lactobacillus* species-specific primers (Pelzer et al., 2018a).

*Lactobacillus* species	Primer name	Primer sequence
*L*. *acidophilus*	LAA-1	5′CATCCAGTGCAAACCTAAGAG3′
	LAA-2	5′GATCCGCTTGCCTTCGCA3′
*L*. *crispatus*	LcrisF	5′AGCGAGCGGAACTAACAGATTTAC3′
	LcrisR	5′AGCTGATCATGCGATCTGCTT3′
*L*. *gasseri*	LgassF	5′AGCGAGCTTGCCTAGATGAATTTG3′
	LgassR	5′TCTTTTAAACTCTAGACATGCGTC3′
*L*. *jensenii*	LjensF	5′AAGTCGAGCGAGCTTGCCTATAGA3′
	LjensR	5′CTTCTTTCATGCGAAAGTAGC3′
*L*. *iners*	InersF	5′GTCTGCCTTGAAGATCGG3′
	InersR	5′ACAGTTGATAGGCATCATC3′

### Taxonomic Classification

Sequence clustering and operational taxonomic unit (OTU) selection was performed using a modified version of CD-HIT-OTU-454 which does not remove singleton clusters (Liu et al., 2011). Taxonomy was assigned to representative sequences by comparison to the latest build of the Greengenes database^2^ using BLAST^3^, and OTU tables were constructed from the output using a custom Perl script (McDonald et al., 2012).

### *Lactobacillus* Phylogenetic Trees

Full-length 16S rRNA sequences for *Lactobacillus* spp. (accession numbers: AB680529.1, AB690249.1, AB668940.1.1, AB008203.1.1, AB425941.1.1, AB008206.1, AF243169.1, AF243167.1, CP018809.253324, CP018809.1516019, CP018809.1347636, CP018809.500868, AB547127.1, AB517146.1, AB932527.1, AB008209.1, HZ485829.7, LG085736.7, LF134126.7, LG104504.7), *Pediococcus pentosaceus* (accession numbers: AB018215.1 and AB362987.1), and *Bacillus subtilis* (accession numbers: AP012496.9810 and AP012496.30276) were downloaded from the SILVA database using the web interface. Sequences were aligned using ClustalW (Thompson et al., 1994) with the default settings. MEGA7 (Kumar et al., 2016) was used to generate the best-known maximum likelihood (ML) tree using a Jukes-Cantor model and 1000 bootstrapping iterations. The ML tree was visualized and edited in FigTree^4^ and Adobe Illustrator (Figure 1).

**FIGURE 2:**
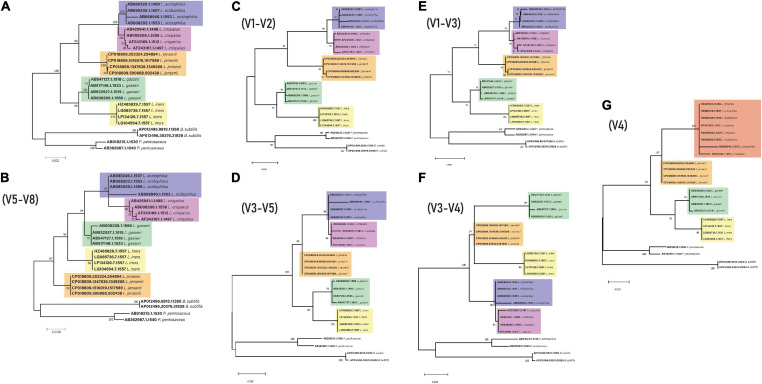
16S rRNA gene phylogeny for key genital tract lactobacilli **(A)** Full length **(B)** V5-V8 region **(C)** V1–V2 region **(D)** V3–V5 region **(E)** V1–V3 region **(F)** V3–V4 region **(G)** V4 – The different color for the top clade is due to the mixed *L. acidophilus* and *L. crispatus* sequences.

To generate the phylogenetic trees for the specific regions, the same full-length 16S rRNA sequences from above were imported into Geneious^5^ along with two *Escherichia coli* sequences downloaded from SILVA (see text footnote 1) (accession number: AB045730.1 and AB045731.1). Sequences were aligned using standard Geneious alignment and trimmed to include the specific variable regions (V1–V2, V1–V3, V3–V4, V3–V4, V4, and V5–V8) using *E. coli* sequences. Trimmed sequences were then aligned using ClustalW with default settings and imported into MEGA7, and trees were constructed and edited in the same way as above (Figure 2).

### Hierarchical Clustering

A dissimilarity matrix was generated based on the relative abundances of *Lactobacillus* spp. in the pyrosequenced and qPCR analyzed samples using the vegdist function in the vegan package in R^6^ with the Bray-Curtis dissimilarity metric (Oksanen et al., 2009). Hierarchical clustering was performed using the hclust function in R with “average” linkage (UPGMA) (R Core Team, 2014). Clustering and relative abundances were visualized in a heatmap with associated dendrogram using the heatmap.2 function from the R package ggplots (Wickham, 2016).

### Re-analysis of Published Endometrial 16S rRNA Sequencing Datasets

Four datasets (Table 3) were re-analyzed using the standard QIIME2^7^ (Bolyen et al., 2019) data analysis pipeline to determine *Lactobacillus* abundance using a standard full-length taxonomic classifier. Each primer pair was used to create *in silico* 16S rRNA gene read libraries using custom databases with the five *Lactobacillus* species of interest (downloaded from SILVA) using Grinder (Angly et al., 2012)^8^, a sequencing simulator. Sequence coverage (the percentages of reads amplified by the primers) was compared between different primer pairs. Each primer pair was used with the custom database for all *Lactobacillus* species (*n* = 1371) to create fastq files with approximately 20,000 sequencing reads. The proportion of reads belonging to each species was identified for each primer and compared to the predicted amplification from the unbiased total dataset. These values correspond to the first column (number of reads) and second column (percentage of reads for each species) within the results table for each primer pair. The proportion of species that was expected to be amplified was compared to the actual amplification predicted by each primer set using custom scripts in R. Primer pairs were also used to *in silico* amplify the human genome to predict low biomass contamination by host DNA using MFEprimer software^9^ (Qu et al., 2009). All bioinformatic scripts used in this study can be found at the following link^10^.

**TABLE 3:** Comparison of publicly available 16S rRNA endometrial datasets used for the sequencing re-analysis.

Dataset	Sample cohort	Sequencing	Primers	Analysis	Total	Total *Lactobacillus* abundance
PRJNA329174 (Moreno et al., 2016)	Endometrial fluid from fertile women at distinct time points before and during pregnancy	454 pyrosequencing	V3–V5_a_	Identified 191 OTUs and stratified samples as either *Lactobacillus* dominant or non-*Lactobacillus* dominant. The non-*Lactobacillus* dominant endometrium was correlated with decreases in implantation, ongoing pregnancy, and live birth rates	2126	248 11.66%
PRJNA546137 (Hernandes et al., 2020)	Vaginal fluid, healthy endometrium and (endometriosis—deep lesions only)	Illumina single end	V3–V4_b_	No significant differences between control and endometriosis patients but did identify that deep endometriotic lesions are correlated with a *Lactobacillus* lacking environment	348	32 9.19%
PRJEB18626 (Wee et al., 2018)	Vaginal, cervical, and endometrial samples of control and women with a history of infertility	Illumina paired end 300	V1–V3_c_	Communities with dominant *L. crispatus* and *L. iners* were identified	55	0%
PRJNA543861 (Winters et al., 2019)	Mid endometrial samples from women undergoing a hysterectomy and compared with other parts of the reproductive tract	Illumina 500 paired end	V4_d_	Concluded that the endometrium is not *Lactobacillus* dominated but is comprised of *Acinetobacter*, *Pseudomonas*, *Cloacibacterium* species	316	2 0.63%

*^*a*^(F342–926) >Forward CCTACGGGAGGCAGCAG >Reverse CCGTCAATTYMTTTRAGT.^*b*^(341–806) >Forward CCTACGGGRSGCAGCAG >Reverse GGACTACHVGGGTWTCTAAT.^*c*^(27–534) >Forward AGAGTTTGATYMTGGCTCA >Reverse TTACCGCGGCTGCTGGCA.^*d*^(515–806) >Forward GTGCCAGCMGCCGCGGTAA >Reverse GGACTACHVGGGTWTCTAAT.*

## Results

### 16S rRNA Amplicon Sequencing Resolution of Genital Tract *Lactobacillus* sp. Operational Taxonomic Units (OTUs) to Genus and Species

In order to determine the *Lactobacillus* species present in upper genital tract (UGT) clinical samples (Table 1), 16S rRNA gene sequencing was performed on the V5-V8 variable region. Ten operational taxonomic units (OTUs) identified in the genital tract were attributable to *Lactobacillus* sp. [*Lactobacillus* sp. genus level (*n* = 2), *L*. *crispatus* (*n* = 2), *L*. *iners* (*n* = 3), *L*. *intestinalis* (*n* = 1), *L*. *jensenii* (*n* = 1), and *L*. *vaginalis* (*n* = 1)]. The majority of *Lactobacillus* sp. OTUs (8/10) were resolved to the genus and species level exploiting the V5–V8 regions of the 16S rRNA gene (Pelzer et al., 2018a).

### *Lactobacillus* Species-Specific Quantitative Real-Time PCR Assay Comparison to 16S rRNA Amplicon Sequencing Output

To confirm the predicted species taxonomic assignment from the 16S rRNA amplicon sequencing output, species-specific quantitative real-time PCR assays were used to confirm the identity and relative abundance of only five of the most prevalent species in the UGT (*L*. *crispatus*, *L. gasseri, L. iners, L. jensenii*, and *L. acidophilus)* in comparison to a standard curve.

**FIGURE 3:**
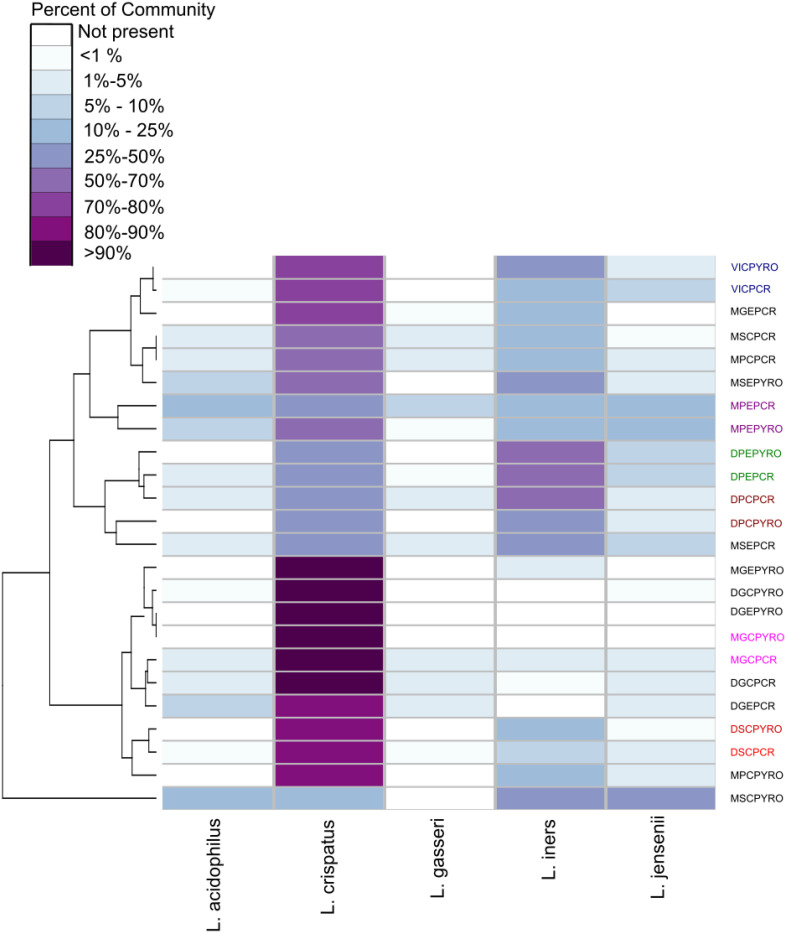
Hierarchical clustering/distance ordination quantifying similarities between qPCR and NGS pairs. *Lactobacillus* relative abundance distribution and hierarchical clustering in NGS and qPCR analyzed samples. The heatmap shows relative abundances of detected *Lactobacillus* species in each sample, with columns organized by relative positions in the dendrogram and grouped by condition being investigated (M, menorrhagia; D, dysmenorrhea; virgo intacta, V), menstrual cycle phase (M, menstrual; secretory, S; proliferative, P; progestin effect, G), and sampling site (endocervix, C; endometrium, E). Some subsets of paired NGS and qPCR samples have been colored to highlight clustering. The sample abbreviations are comprised of two parts: the clinical information and the molecular technique (PYRO or qPCR). The clinical abbreviations can be found in Table 1.

The abundance of the five most prevalent species (*L. crispatus, L. gasseri, L. iners, L. jensenii*, and *L. acidophilus*) were then compared between qPCR and relative abundances as determined by amplicon sequencing (Figure 3). *L. crispatus and L. iners* predominated the *Lactobacillus* community in most samples when characterized by 16S rRNA amplicon sequencing. All samples displayed similar abundance profiles with enriched lower abundance species including *L. jensenii, L. gasseri*, *L. iners*, and *L. acidophilus* detected by qPCR (Figure 3 and Supplementary Table 2). As shown in Figure 3, *L*. *crispatus* and *L. iners* predominated the microbial communities in samples analyzed in this study and samples were more likely to cluster based on *Lactobacillus* community predominance, than the patient history (dysmenorrhea or menorrhagia), the anatomical site of collection (endometrium or cervix), or the analysis technique (qPCR or NGS).

### *Lactobacillus* Phylogeny

In order to understand if the use of different variable regions of the 16S rRNA gene results in changes to taxonomic classification, phylogenetic trees were constructed using the full-length 16S rRNA gene sequences of the *Lactobacillus* sp. described in this study. These trees indicated that *L*. *acidophilus* and L. *crispatus* appear as sister groups derived from a single node for all primer pairs except V4, where the two species appear as a single group of descendent taxa (Figure 2).

### Primer Pair Comparison Using Published Endometrial 16S rRNA Sequencing Data

**FIGURE 4:**
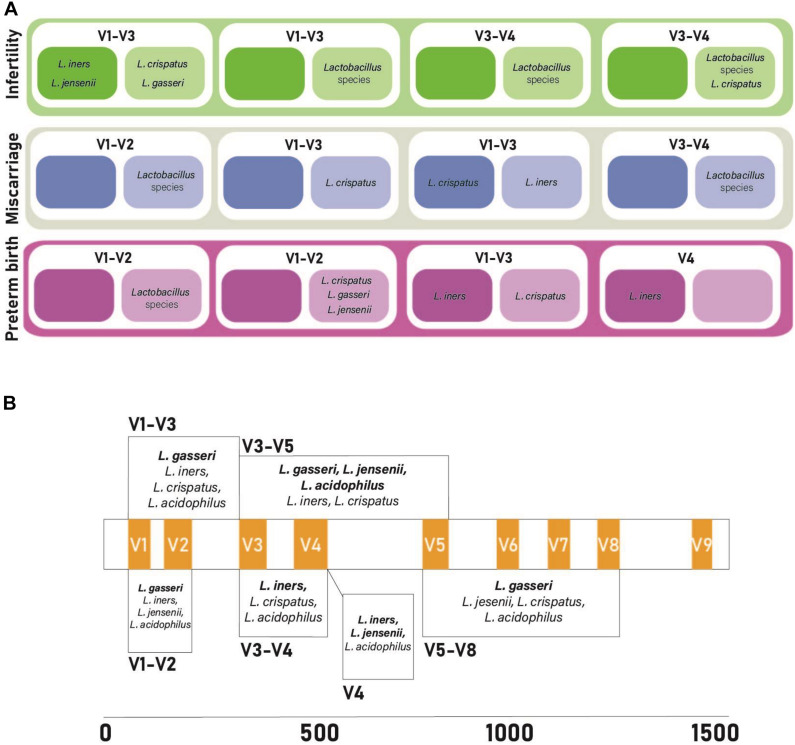
Comparison of primer bias and findings from previously published vaginal microbiome studies investigating miscarriage, infertility, and preterm birth. **(A)** 16S rRNA primer pairs amplifying vaginal lactobacilli. Darker colored boxes represent increased abundance of lactobacilli, lighter colored boxes represent decreased abundance of lactobacilli in cases of infertility, miscarriage, and pre-term birth. **(B)** Predictive bias of primer pairs along the 16S rRNA gene. Hypervariable regions appear in orange. Each box is the predictive bias of the primer pairs that it spans. Bolded bacterial species names correlate with under-reported lactobacilli abundance and non-bolded bacterial species names correlate with over-reported lactobacilli abundance. References for the data from studies included in this figure can be found in Supplementary Table 1.

To determine if alternative regions of the 16S rRNA gene lead to changes for *Lactobacillus* species prediction, publicly available datasets with different primer pairs were used for *Lactobacillus* species comparisons, primer biasing analysis, and host DNA amplification prediction. All four published datasets used variable region primer pairs targeting different regions of the 16S rRNA gene (Table 3). The total predicted abundance of *Lactobacillus* sp. varied from 11.66% (V3–V5) and 9.19% (V3–V4) to 0.63% (V4) and 0% (V1–V3). Of the four datasets, three were Illumina technologies and two of these were paired-end studies (300 base pair and 500 base pair). However, despite the differences in experimental design, datasets were independently classified into amplicon sequence variants (ASVs) and then used for taxonomic analysis to allow comparison of results. OTUs were used for the original data analysis in our earlier publications characterizing the upper genital tract microbiome. Therefore, we retained the data in OTU format for consistency and included it in the original form in this manuscript. ASVs were used in the re-analysis of the four published datasets because that is now the accepted method of taxonomic classification. The two methods of analysis were not ever directly compared because OTUs are a combination of similar sequences and ASVs are not. None of the *Lactobacillus* sp. of interest in this study were speciated using the standard QIIME2 taxonomic classifier. Sequence coverage differences between primer pairs for the simulated control dataset was statistically significant (*p* = 2e-16). Potential primer sequencing bias was identified for all primers in the study, including the V5-V8 primers used by our group (Table 4 and Figure 4). All primer pairs inaccurately reported the abundance of *Lactobacillus* sp. in the lower genital tract. All primer pairs except for those targeting the V4 region had predicted human genome amplification (data not shown).

**TABLE 4:** Predicted *Lactobacillus* species amplification using *in silico* PCR.

	Database		Primer pairs (total # of reads that were attributed to each species from the 20000 and the percentage of total reads that were from each species)											
Species	Total #	%	V1–V2^A^		V3–V5^B^		V3–V4^C^		V1–V3^D^		V4^E^		V5-V8	
*L. gasseri*	635	*46.31656*	6,470	32.35	5,790	28.96448	5,851	29.255	6,204	31.02	8,512	42.56	5,529	27.645
*L. iners*	76	*5.543399*	1,821	9.105	1,283	6.418209	1,185	5.925	1,936	9.68	9,68	4.84	1,084	5.42
*L. crispatus*	311	*22.68417*	5,031	25.155	5,341	26.71836	5,753	28.765	5,027	25.135	4,638	23.19	5,704	28.52
*L. jensenii*	82	*5.981036*	1,918	9.59	1,423	7.118559	1,275	6.375	1,919	9.595	1,095	5.475	1,411	7.055
*L. acidophilus*	267	*19.47484*	4,760	23.8	6,153	30.78039	5,936	29.68	4,914	24.57	4,787	23.935	6,272	31.36
Total	1,371	100%	20,000	100%	19,990	100%	20,000	100%	20,000	100%	20,000	100%	20,000	100%

*^*A*^(28–388) >Forward GAGTTTGATCNTGGCTCAG >Reverse TGCTGCCTCCCGTAGGAGT.^*B*^(F342–926) >Forward CCTACGGGAGGCAGCAG >Reverse CCGTCAATTYMTTTRAGT.^*C*^(341–806) >Forward CCTACGGGRSGCAGCAG >Reverse GGACTACHVGGGTWTCTAAT.^*D*^(27–534) >Forward AGAGTTTGATYMTGGCTCA >Reverse TTACCGCGGCTGCTGGCA.^*E*^(515–806) >Forward GTGCCAGCMGCCGCGGTAA >Reverse GGACTACHVGGGTWTCTAAT.*

## Discussion

This study examined the bacterial composition of the communities in the upper genital tract of women, reporting changes in the bacterial community composition of lactobacilli, revealing that the primer selection may significantly bias the predicted microbial community profile. Importantly, 16S rRNA primer pairs used to characterize the low-biomass upper genital tract should be selected to most accurately amplify targeted lactobacilli. Sequencing of specific variable regions of the 16S rRNA gene improves the discriminatory power for speciation of dominant genital tract lactobacilli (Graspeuntner et al., 2018). Further, there exists a compromise in primer selection for correct reporting of taxonomic classification and species abundance and low host genome amplification for low-biomass sites. Current studies may select primers that have low host genome amplification, such as the V4 region, but at the cost of reduced sensitivity in differentiating species such as *L. acidophilus* and *L. crispatus* (evidenced in Figure 2). There also exists a growing body of evidence highlighting the cross-amplification of host DNA using 16S rRNA primers (Walker et al., 2020).

Sequencing technologies frequently only report the presence of lactobacilli at the genus level. Studies exploiting some regions of the 16S rRNA gene fail to discriminate lactobacilli beyond higher order taxonomic classification due to limited sequence variation (Brown et al., 2018; Wee et al., 2018; Bernabeu et al., 2019; Zhang et al., 2019; Al-Memar et al., 2020; Tu et al., 2020). Therefore, some studies have reported that lactobacilli in the lower genital tract as a genera are: positively correlated with healthy pregnancy outcomes including successful implantation and delivery at term; and form abundant community members in cases of infertility, adverse pregnancy outcomes including recurrent implantation failure and preterm birth, and cancer (Moreno et al., 2016; Chen et al., 2017; Miles et al., 2017). For example, *L. iners* in the lower genital tract reportedly does not protect against preterm birth and is frequently reported as an abundant community member in women with bacterial vaginosis suggesting that the presence of any *Lactobacillus* species is not indicative of a health milieu (Madhivanan et al., 2014; Petricevic et al., 2014). In addition, our data suggest that detection of *L. iners* by 16S rRNA amplicon sequencing may represent an overestimation of this species. Further, significant differences between lactobacilli in term compared to preterm deliveries have not been reported for all studies (Romero et al., 2014; Amabebe and Anumba, 2018).

Examination of the genital tract as a continuum concluded that sampling high-biomass lower genital tract sites including the vagina or cervix identified distinct microbial community profiles compared to more diverse, but low-biomass upper genital tract sites including the endometrial cavity, fallopian tubes, or peritoneal cavity (Chen et al., 2017; Pelzer et al., 2018a,b), perhaps highlighting the need for more discriminatory analyses to tease out significant taxa associated with the genital tract sites in health and disease.

*L. crispatus* and *L. iners* were the most abundant lactobacilli in the samples tested by 16S rRNA amplicon sequencing in our study, which is consistent with the previous studies that we re-analyzed as part of this project. We observed the same shortfalls commonly associated with lower order taxonomic classification and low-biomass samples. Similar results can be observed in molecular studies characterizing the female genital tract when multiple variable regions of the 16S rRNA gene were sequenced (Fettweis et al., 2012; Madhivanan et al., 2014; Miles et al., 2017; Graspeuntner et al., 2018). Van Der Pol et al. (2019) reported that the choice of 16S rRNA reference sequence database and sample sequence clustering parameters are equally as important as the choice of variable region for amplification characterizing microbial community members to lower orders.

There is no doubt that sequencing the conserved 16S rRNA gene has improved our understanding of extant biodiversity in human microbial communities and is critical for understanding the impact of low-abundance community members on health and disease. However, there is no consensus best practice for microbiome studies of the female genital tract, and significant variability exists between sample collection and storage methods, DNA extraction, universal primer selection, and sequencing platform and data analysis software (Pollock et al., 2018). Characterization of microbial communities using the 16S rRNA gene have been hampered by inherent differences generated in community profiles when sequencing different hypervariable regions, short read lengths, and taxonomic classification difficulties due to limited resolution for closely related species (Poretsky et al., 2014). Sequencing technologies have been used to interrogate the genital tract microbial community in reproductive-age women but most fail to resolve the isolates to the species level. The V4 region is favored because amplification does not result in non-specific host DNA amplification, however, as demonstrated by phylogenetic analyses, the ability to confidently resolve lower order taxonomic classification for lactobacilli is a trade-off in genital tract analyses. However, Lactobacilliales is a diverse and heterogeneous family with some members genetically very closely related, but lacking a clear monophyletic origin, impeding the ability to accurately identify lower order taxonomy in studies exploiting conserved genes such as the 16S rRNA gene (Heilig et al., 2002).

Consequently, more recent efforts have focused on sequencing multiple variable regions of the gene with amalgamation of all data into a single profile (Fuks et al., 2018). The most recent research focuses on removing bias associated with sequencing component variable regions by using full-length gene sequencing (Callahan et al., 2016). The need to characterize the full-length 16S rRNA gene is further required as exhibited by the change in *L. gasseri, L. jensenii*, and *L. iners* clustering when comparing the full-length gene to specific variable regions. The ability to confidently assign the correct identity to key genital tract lactobacilli is hampered by primer selection.

Collectively our research confirms what other studies have shown, that health and disease (Figure 4) may depend on species and strain-level differences for prominent community members at a given anatomical niche (Kraal et al., 2014). It is clear that additional discriminatory power is required to resolve lower order taxonomic classifications using current sequencing methods. This report confirms that speciation of key genital tract *Lactobacillus* sp., capable of modulating reproductive health is possible when the appropriate region of the 16S rRNA gene is interrogated.

## Conclusion

Studies characterizing microbial communities in the female genital tract report inconsistent results when assessing dysbiosis as a cause of reproductive pathology. This paper provides further evidence for the impact of primer selection on evaluating the biological significance of shifts in community taxa in the endometrial cavity. Careful experimental design should include a comparative analysis of microbial community profiling data generated by interrogation of multiple variable regions to the 16S rRNA gene or the full-length gene to ensure that species abundance and diversity are accurately reflected.

## Data Availability Statement

The datasets presented in this study can be found in online repositories. The names of the repository/repositories and accession number(s) can be found in the article/Supplementary Material.

## Ethics Statement

The studies involving human participants were reviewed and approved by the UnitingCare Health Human Research Ethics Committee and Queensland University of Technology Human Research Ethics Committee. The patients/participants provided their written informed consent to participate in this study.

## Author Contributions

JO’C and DW designed and completed bioinformatics analyses, contributed to the analysis and interpretation of the data, and contributed to the writing of the manuscript. MB conceived and designed the project, performed collection of clinical specimens, and contributed to the writing of the manuscript. FH designed and completed the qPCR experiments, contributed to the analysis and interpretation of the qPCR data, and contributed to the writing of the manuscript. EP conceived and designed the project, completed tissue processing, DNA extraction, and 16S PCR experiments, contributed to the analysis and interpretation of the data, and drafted significant parts of the work. All authors contributed to the article and approved the submitted version.

## Conflict of Interest

The authors declare that the research was conducted in the absence of any commercial or financial relationships that could be construed as a potential conflict of interest.

## Publisher’s Note

All claims expressed in this article are solely those of the authors and do not necessarily represent those of their affiliated organizations, or those of the publisher, the editors and the reviewers. Any product that may be evaluated in this article, or claim that may be made by its manufacturer, is not guaranteed or endorsed by the publisher.

## Footnotes

1
https://www.arb-silva.de/

2
https://greengenes.secondgenome.com/

3
https://blast.ncbi.nlm.nih.gov/Blast.cgi

4
http://tree.bio.ed.ac.uk

5
https://www.geneious.com/

6
https://www.r-project.org/

7
http://qiime.org/

8
https://sourceforge.net/projects/grinder/

9
https://www.mfeprimer.com/

10
https://github.com/jessicaocallaghan/primer_analysis_16S_v_regions

## Abbreviations

ATCC: American type culture collection
DGC: dysmenorrhea progestin effect endocervix
DGE: dysmenorrhea progestin effect endometrium
DNA: deoxyribonucleic acid
DPC: dysmenorrhea proliferative endocervix
DPE: dysmenorrhea proliferative endometrium
DSC: dysmenorrhea secretory endocervix
DSE: dysmenorrhea secretory endometrium
HRM: high resolution melt
MGC: menorrhagia progestin effect endocervix
MGE: menorrhagia progestin effect endometrium
MPC: menorrhagia proliferative endocervix
MPE: menorrhagia proliferative endometrium
MSC: menorrhagia secretory endocervix
MSE: menorrhagia secretory endometrium
OUT: operational taxonomic unit
rRNA: ribosomal ribonucleic acid
VIC: virgo intacta.
