# Supplementary material for: Limitations of 16S rRNA Gene Sequencing to Characterize Lactobacillus Species in the Upper Genital Tract
Source: Front Cell Dev Biol. 2021 Jul 29;9:641921. doi: 10.3389/fcell.2021.641921 (PMC8359668; doi:10.3389/fcell.2021.641921)
Supplement: Supplementary file 1 [file Data_Sheet_1.docx]

| **Supplementary Table 1:** | | | **Findings ^A^** | |  |
| --- | --- | --- | --- | --- | --- |
| **Condition** | **Primer Pair** | **Specific comparison** | **Increased abundance** | **Decreased abundance** | **Reference** |
| Infertility | V3-V4 | PCOS and healthy women of reproductive age |  | *Lactobacillus* spp | (7) |
|  | V1-V3 | Infertile women and fertile women | *L. iners, L. crispatus* | *L. gasseri* | (41) |
|  | V1-V3 | Women who have or had infertility and women with no history of infertility |  | *Lactobacillus* spp | (8) |
|  | V3-V4 | Patients undergoing assisted reproductive treatment compared women who achieved pregnancy and those who did not |  | *Lactobacillus* spp  (*L. crispatus*) | (9) |
| Miscarriage | V1-V3 | Women who are not currently pregnant and a subgroup with previous association with miscarriage or abortion | *L. crispatus* |  | (42) |
|  | V1-V2 | Women who had had either first trimester miscarriage, second trimester miscarriage and term births compared using results from 5 weeks gestation |  | *Lactobacillus* spp | (10) |
|  | V3-V4 | Women with recurrent miscarriage and to control women (no history of miscarriage) |  | *Lactobacillus* spp | (11) |
|  | V1-V3 | First trimester (8-12 weeks) pregnancies and the subsequent outcomes | *L. crispatus* | *L. iners* | (43) |
| Preterm birth | V1-V2 | Early pregnancy between 8 -12 weeks with and without risk factors for preterm birth |  | *Lactobacillus* spp | (12) |
|  | V1-V3 | Women at approximately 16 weeks gestation and between women who had preterm birth and those who did not | *L. iners* | *L. crispatus* | (44) |
|  | V1-V9 | Women who did and did not have risk factors and correlated with subsequent preterm birth |  | *L. crispatus* | (45) |
|  | V1-V2 | Women in early pregnancy (between 6 and 14 weeks) of women with and without preterm birth risk factors |  | *L. gasseri, L. iners, L. crispatus, L. jensenii* | (46) |
|  | V4 | Women who had a preterm birth compared to healthy term birth | *L. iners* |  | (47) |

**^A^** – Comparative abundance for the condition that is being measured (i.e. Increased and decreased abundance in infertile women)

| **Supplementary Table 2: Species-specific qPCR** | | | | | | | | | |
| --- | --- | --- | --- | --- | --- | --- | --- | --- | --- |
| *L. acidophilus* | | *L. gasseri* | | *L. crispatus* | | *L. jensenii* | | *L. iners* | |
| Sample | ng/µl | Sample | ng/µl | Sample | ng/µl | Sample | ng/µl | Sample | ng/µl |
| DGC | 0.0058 | DGC | 0 | DGC | 0.0002 | DGC | 0 | DGC | 0 |
| DSG | 0.0152 | DSG | 0.0001 | DSG | 0 | DSG | 0.0009 | DSG | 0.0032 |
| MGC | 0.9375 | MGC | 1.0293 | MGC | 0.6446 | MGC | 0 | MGC | 0 |
| DPC | 0.0110 | DPC | 0.0005 | DPC | 0 | DPC | 0 | DPC | 0 |
| DGE | 0.3461 | DGE | 0.0001 | DGE | 0 | DGE | 0.1437 | DGE | 0 |
| DPE | 0.0582 | DPE | 0.0622 | DPE | 0.0494 | DPE | 0 | DPE | 0 |
| MGE | 0.0121 | MGE | 0.0022 | MGE | 0.0025 | MGE | 0 | MGE | 0 |
| MSE | 0.2557 | MSE | 0 | MSE | 0 | MSE | 0.0160 | MSE | 0 |
| MSC | 0.0745 | MSC | 0 | MSC | 0.0036 | MSC | 0.0021 | MSC | 0.0146 |
| MPE | 0.9266 | MPE | 0.0385 | MPE | 0.0259 | MPE | 0 | MPE | 0.0023 |
| MPC | 0.6556 | MPC | 0 | MPC | 0.0005 | MPC | 0.5110 | MPC | 0 |
| VIC | 0.0001 | VIC | 0 | VIC | 0.0098 | VIC | 0.0007 | VIC | 0.0032 |

| Standard | ng/ul) |
| --- | --- |
| *L. gasseri* ATCC | 0.6996 |
| *L. gasseri* ATCC | 0.7952 |
| *L. gasseri* ATCC | 0.0638 |
| *L. gasseri* ATCC | 0.0705 |
| *L. gasseri* ATCC | 0.0075 |
| *L. gasseri* ATCC | 0.0074 |


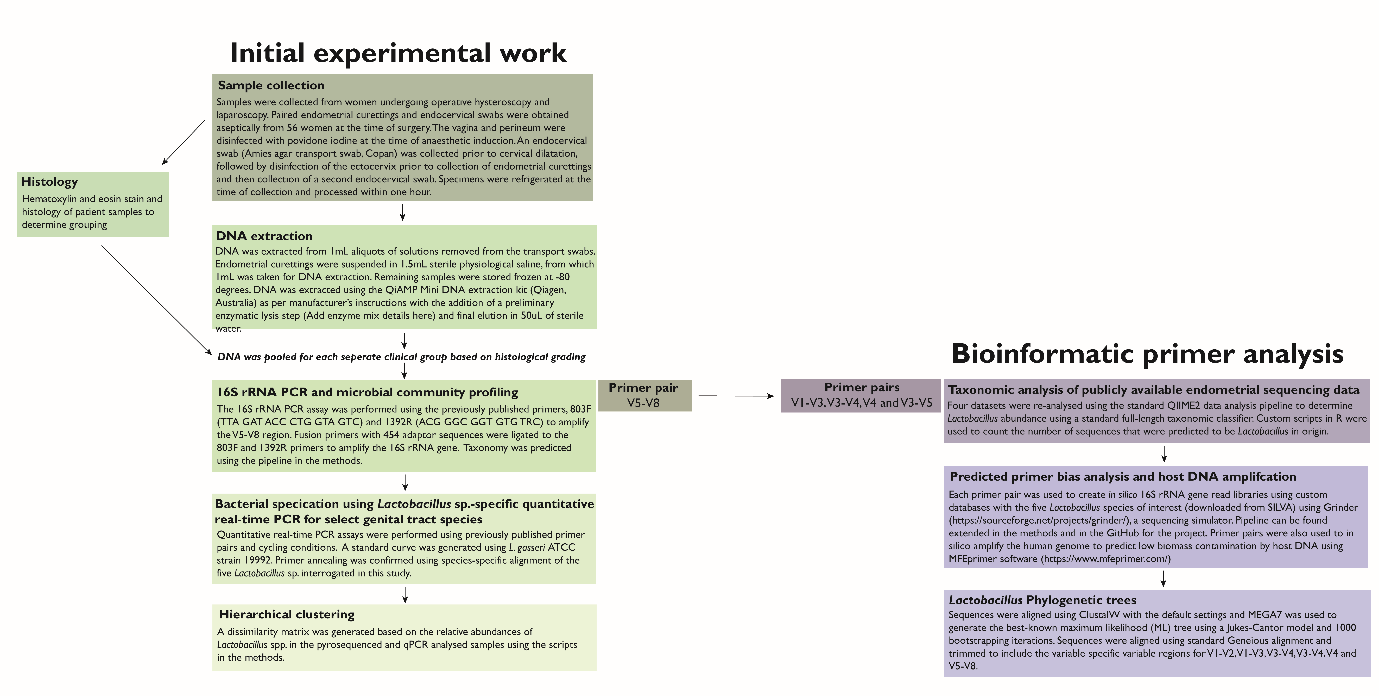


**Supplementary Figure 1**: Methods used in this study (wet laboratory based and bioinformatics). The flowchat demonstrates the logical flow and link from the initial experimental work to the bioinformatic reanalysis of different primers. Extended methods can be found in the corresponding methods section and/or in the GitHub repository for this project.
